# Supplementary material for: Activation of serotonin neurons promotes active persistence in a probabilistic foraging task
Source: Nat Commun. 2018 Mar 8;9:1000. doi: 10.1038/s41467-018-03438-y (PMC5843608; doi:10.1038/s41467-018-03438-y)
Supplement: Supplementary file 1 — Supplementary Information [file 41467_2018_3438_MOESM1_ESM.pdf]

Activation of serotonin neurons promotes active persistence in a probabilistic foraging task

Lottem et al.

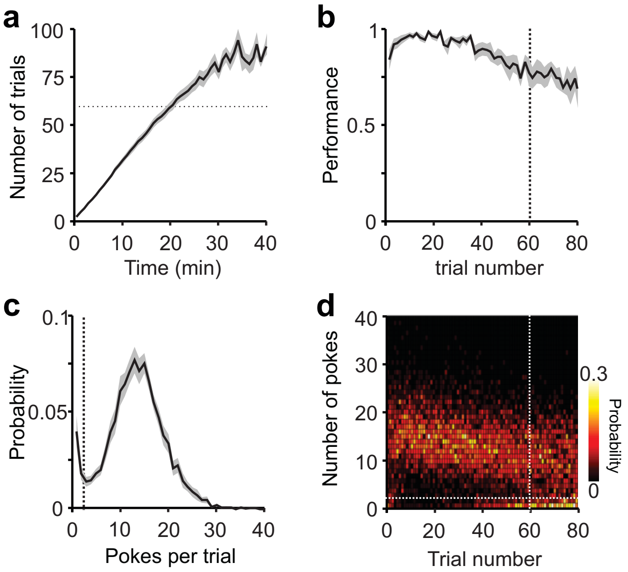

Supplementary figure 1 Task performance and trial selection criteria

(a) Plot showing average cumulative number of trials as a function of time (n = 16). Only trials 1 to 60 were considered for further analysis (cutoff marked by dashed line).

(b) Plot showing average performance, defined as the probability of correctly alternating between reward sides as a function of trial number (cutoff marked by dashed line).

(c) Average poke-per-trial distribution (n = 16). Note the bimodal shape of the distribution. Trials with less than 3 pokes were considered as "lapses" and discarded from further analysis (dashed line).

(d) Hit map showing average poke-per-trial distributions as a function of trial number and the two selection criteria (dashed lines marking trial number and number of pokes).

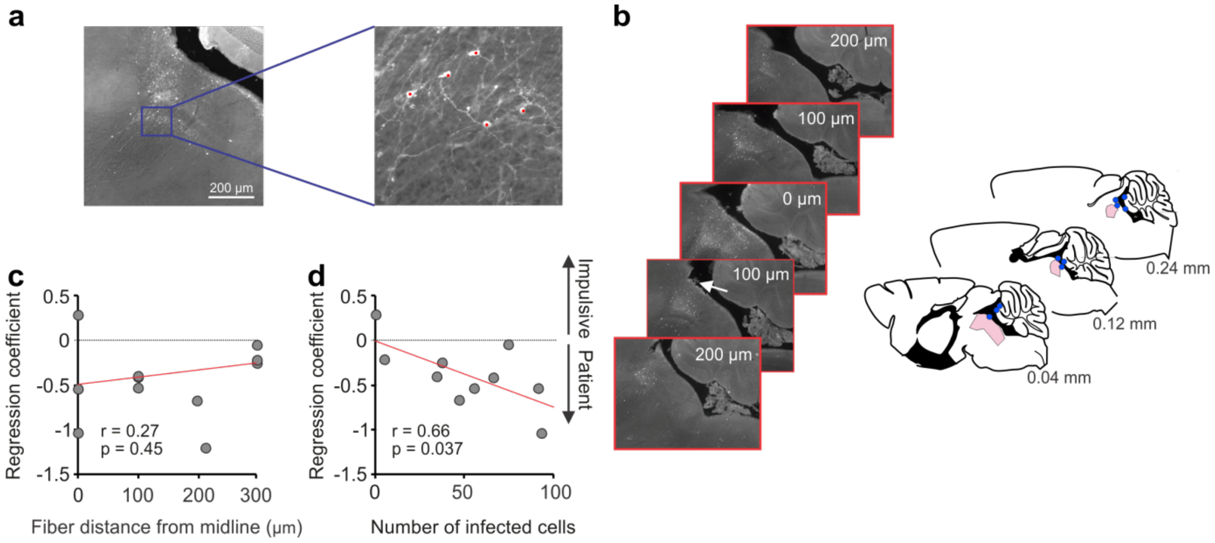

Supplementary figure 2 Histological analysis of virus expression and fiber location

(a) Left: A fluorescence image of a parasagittal section of dorsal raphe nucleus of an example ChR2-YFP expressing, SERT-Cre mouse. Right: Magnification of the same image, red dots mark labeled neurons.

(b) Left: Five parasagittal images taken from a different mouse (adapted with permission from Ref. 63). Numbers indicate distance from midline. White arrow points at the location of the fiber tip. Right: Fiber tip locations for the 10 SERT-Cre mice. Dorsal raphe nucleus is indicated in pink.

(c) Correlation between Cox regression photostimulation coefficient and fiber location for SERT-Cre mice ($n = 10$). The red line is a linear regression curve, with its correlation coefficient shown as well ($p > 0.05$).

(d) Correlation between Cox regression photostimulation coefficient and number of YFP expressing cells for SERT-Cre mice ($n = 10$). The red line is a linear regression curve, with its correlation coefficient shown as well ($p < 0.05$).

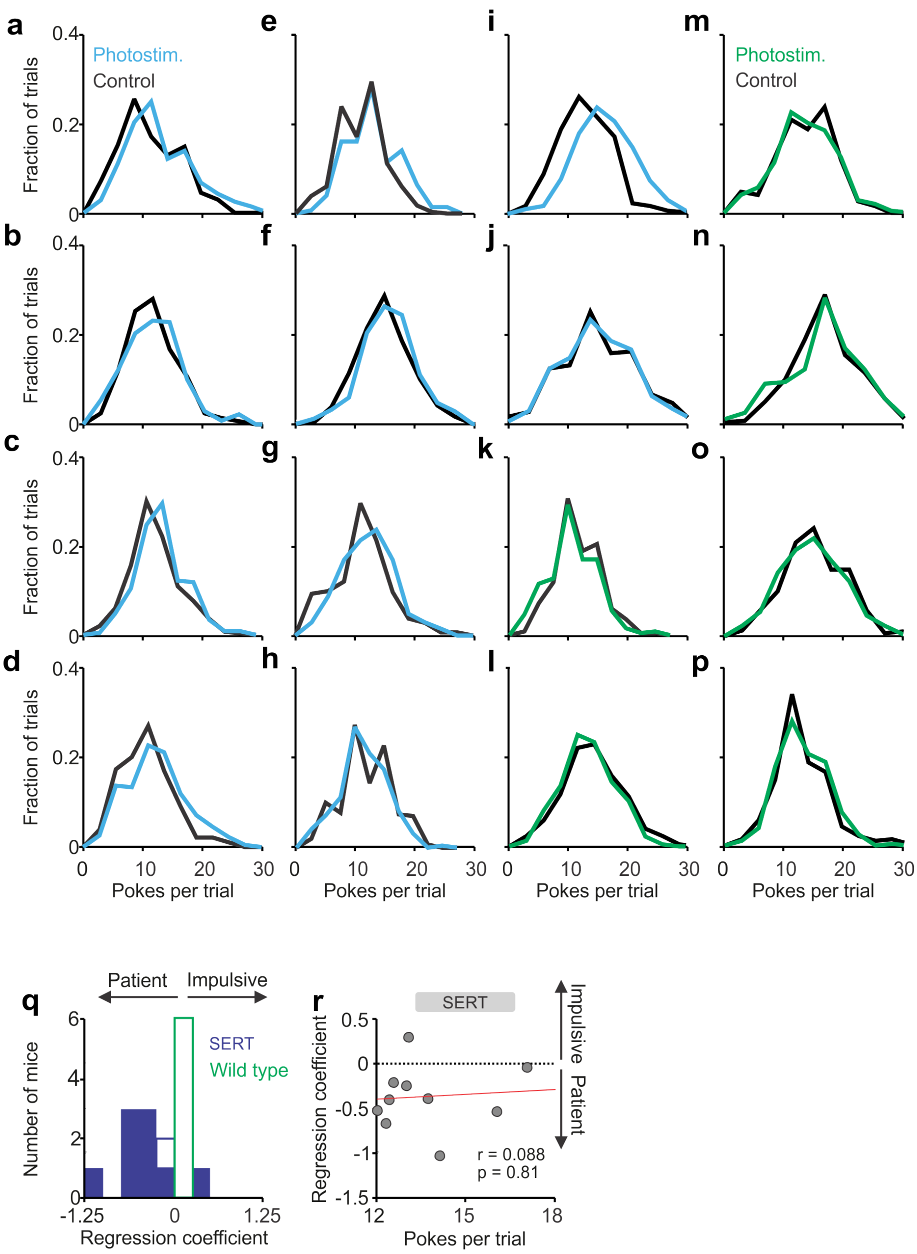

Supplementary figure 3 The effect of DRN 5-HT photostimulation on individual mouse switching behavior

(a-p) Distributions of the number of pokes per trial for photostimulated trials (blue for SERT-Cre mice and green for wild-type) and control trials (black) for all the mice used in this study.

(q) Histogram of Cox regression photostimulation coefficient for SERT-Cre (blue; $n = 10$) and wild-type (green; $n = 6$) mice. Filled bars correspond to significant coefficients ($p < 0.05$).

(r) Correlation between Cox regression photostimulation coefficient and average number of pokes per trial for SERT-Cre mice ($n = 10$). The red line is a linear regression curve, with its correlation coefficient shown as well ($p > 0.05$).

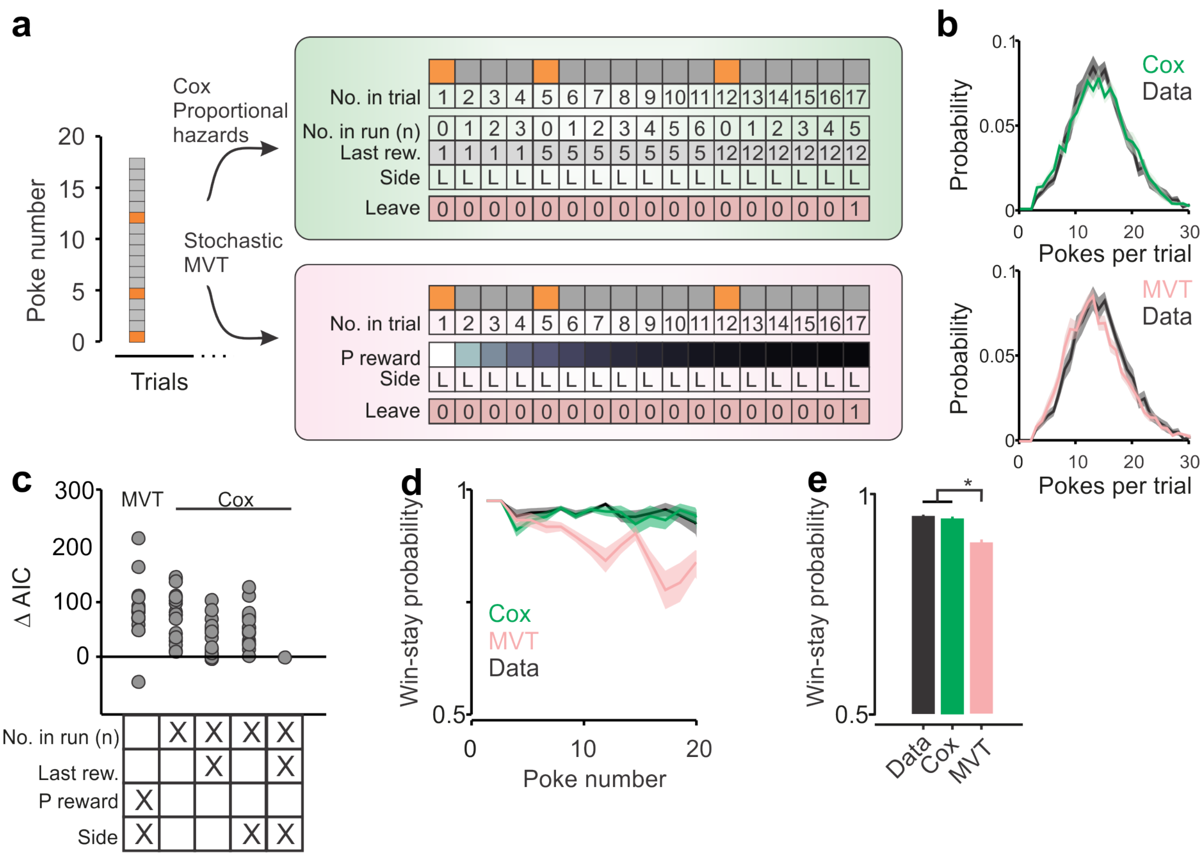

Supplementary figure 4 Model comparison

(a) Schematic drawing of model-fitting pipe-line for either the proportional hazards or stochastic MVT model. Top: in the proportional hazards model, each nose-poke was labeled with a vector of values corresponding to the different Cox regression coefficients. These values, together with the outcome of each poke (stay or leave) were used to fit a logistic regression model - the outcome of which was an estimated hazard rate that is reset at trial start and after each reward, and is multiplicatively changed by the different coefficient values. Bottom: in the stochastic MVT model, the subjective reward probability (calculate using Eq. 2 and depicted here with grayscale coloring) and side were used to fit a logistic regression model for mouse leaving decisions.

(b) Average pokes-per trial distributions for real and simulated data using the proportional hazards model (top; green) and the stochastic MVT model (bottom; pink).

(c) Akaike information criteria (AIC) for the stochastic MVT and four versions of the proportional hazards model. Regressors used in each of the models are indicated in the matrix below (n = 16 mice).

(d) Win-stay probability as a function of poke number within a trial for real and simulated data (n = 16 mice).

(e) Bar plot showing the average win-stay probability for the two models and data (n = 16 mice). $P < 0.05$, ANOVA.

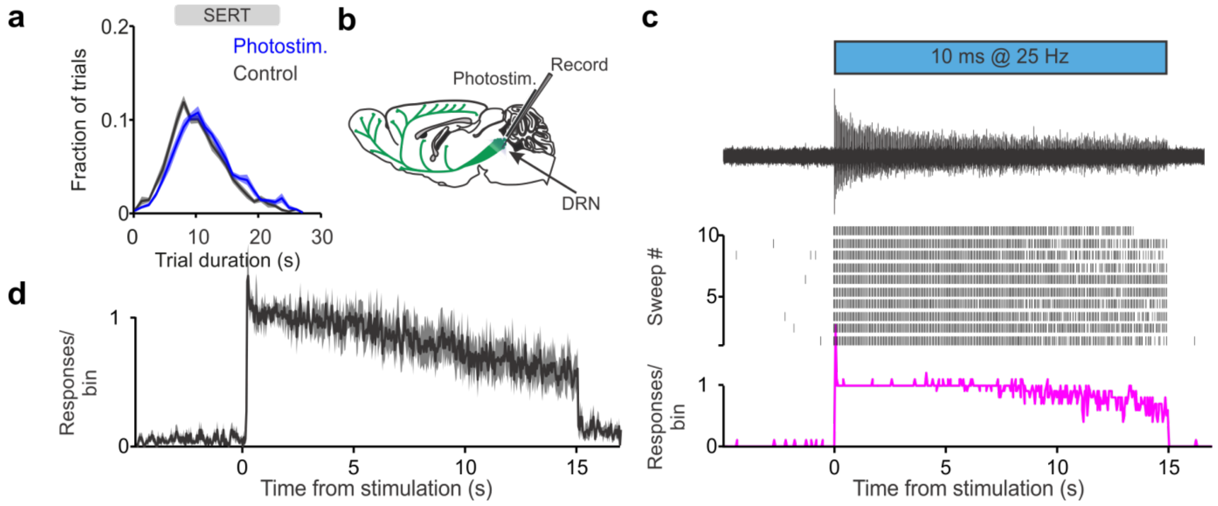

Supplementary figure 5 Electrophysiological validation of DRN 5-HT photostimulation

(a) Distributions of trial durations for photostimulated (blue) and control trials (black) averaged across the population of SERT-Cre mice (n = 10).

(b) Schematic of the experimental setup. Multi-unit responses to light delivery of ChR2-expressing DRN neurons were recorded using an optrode (adapted with permission from Ref. 63).

(c) Top: Example multi-unit response to 15 s, 25 Hz photostimulation. Bottom: Mean responses of the same unit to 10 stimulation sweeps. Responses were binned in 40 ms bins aligned on light-pulse onset.

(d) Average binned multi-unit responses to 15 s, 25 Hz photostimulation (n = 7 multi-units from 2 mice).

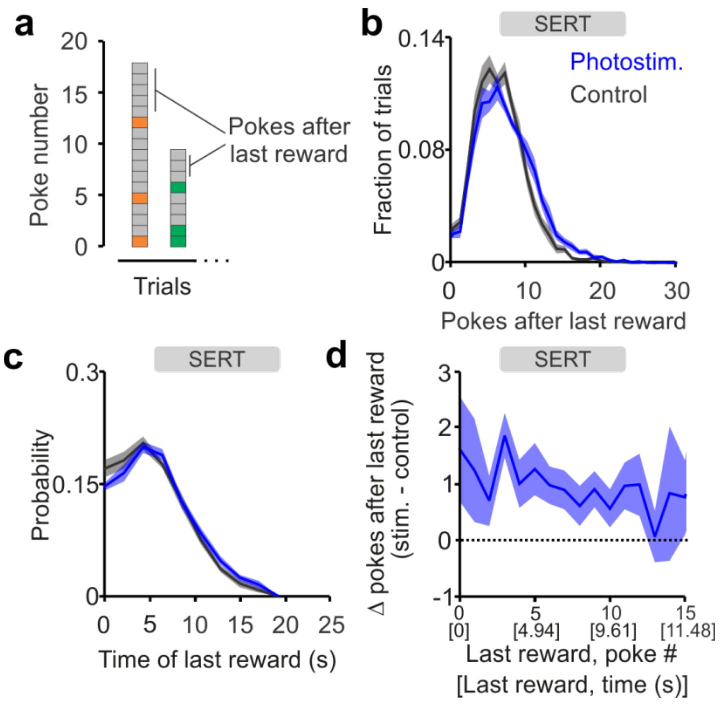

Supplementary Figure 6 The effect of DRN 5-HT photostimulation as a function of its duration

(a) Two consecutive example trials, the first had five pokes after the last reward and the second had three.

(b) Distributions of the number of pokes after last reward for photostimulated trials (blue) and control trials (black) averaged across the population of SERT-Cre mice ($p < 0.05$, Wilcoxon sign-rank test, $n = 10$).

(c) Distributions of last reward times for photostimulated (blue) and control (black) trials across the population of SERT-Cre mice ($p < 0.05$, Wilcoxon sign-rank test, $n = 10$).

(d) Difference between average number of pokes after last reward in photostimulated and control trials as a function of last reward position or time for SERT-Cre mice ($n = 10$). Last reward times are shown in brackets and are the average times of the rewards occurring at the corresponding position. $p > 0.05$, linear regression analysis.

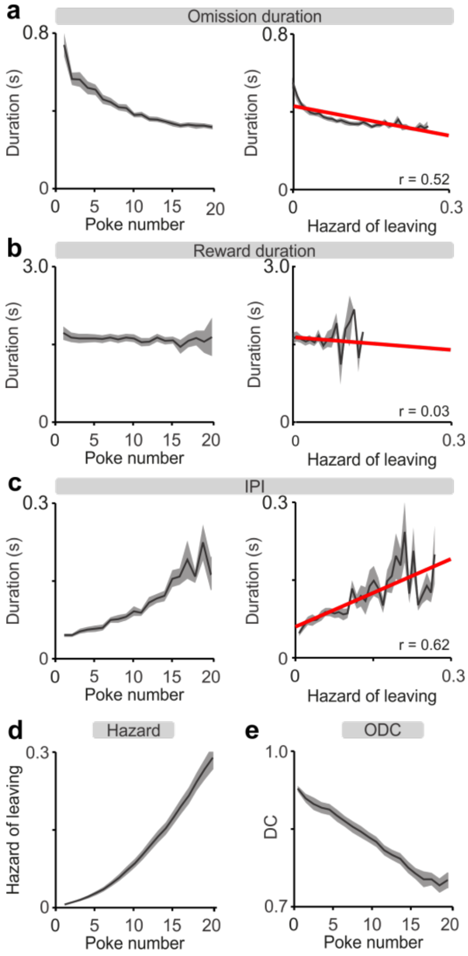

Supplementary figure 7 Correlation between hazard and nose-poking behavior

(a) Left: omission nose-poke duration as a function of nose-poke number within a trial. Right: correlation between omission duration and estimated hazard. The red line is a linear regression curve, with its equation and correlation coefficient shown as well ($p < 0.001$).

(b) Left: Rewarded nose-poke duration as a function of nose-poke number within a trial. Right: correlation between reward duration and estimated hazard. The red line is a linear regression curve, with its equation and correlation coefficient shown as well ($p > 0.05$).

(c) Left: Inter-poke-intervals duration as a function of nose-poke number within a trial. Right: correlation between inter-poke-interval duration and estimated hazard. The red line is a linear regression curve, with its equation and correlation coefficient shown as well ($p < 0.001$).

(d) Estimated hazard as a function of nose-poke number within a trial.

(e) ODC as a function of nose-poke number within a trial.

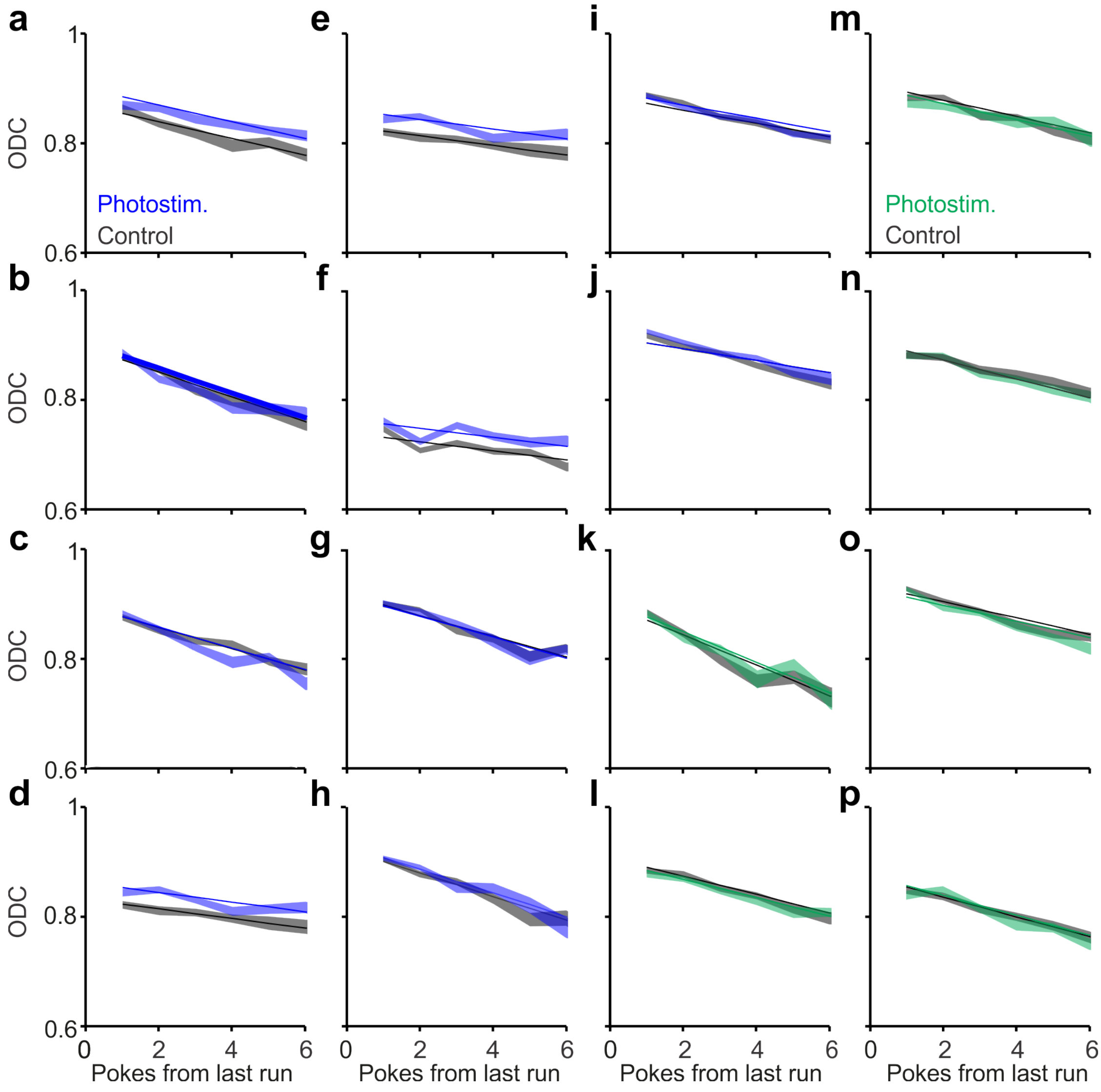

Supplementary figure 8 The effect of DRN 5-HT photostimulation on the microstructure of individual mouse behavior

(a-p) The ODC aligned on last reward for photostimulated trials (blue for SERT-Cre mice and green for wild-types) and control trials (black) for all the mice used in this study. The lines are linear regression curves.
